# Hazards and risk assessment of heavy metals from consumption of locally manufactured painkiller drugs in Nigeria

**DOI:** 10.1016/j.toxrep.2020.08.009

**Published:** 2020-08-21

**Authors:** John Kanayochukwu Nduka, Henrietta Ijeoma Kelle, Emeka Chima Ogoko

**Affiliations:** a Environmental Chemistry and Toxicology Research Unit, Pure and Industrial Chemistry Department, Nnamdi Azikiwe University, P.M.B 5025, Awka, Nigeria; b Department of Pure and Applied Science, Faculty of Sciences, National Open University, Abuja, Nigeria

**Keywords:** Local painkiller drugs, Heavy metals, Carcinogenic and Non carcinogenic effect, Risk assessment, Health risks, Nigeria

## Abstract

In the informal sector of Nigeria’s economy, jobs are done manually as against automation resulting in body stress and pain, hence the need for painkiller drugs. Thirty different locally manufactured painkiller drugs, with analgesic, antipyretic and anti-inflammatory effects, were randomly sampled from pharmaceutical shops within Awka in October 2016. The drugs were pulverised, sieved and ashed before digestion using conc aqua regia HCl : HNO_3_ (3:1), carcinogenic heavy metals (arsenic, cadmium, chromium, mercury, nicked and lead) were assayed using Varian AA240 atomic absorption spectrophotometer (AAS). Risk assessment was carried out using US EPA model. The highest levels of arsenic (0.350 mg/kg) were found in samples with code 01, 03 and 020, cadmium (0.107 mg/kg and 0.144 mg/kg) were in samples code 013 and 028, and samples 03 and 011 had chromium levels as 6.637 mg/kg and 5.298 mg/kg. Highest value of mercury (0.470 mg/kg) was in sample code 01. All the painkiller drugs have nickel in the range of 0.046−0.448 mg/kg while highest values of lead were in sample code 05, 025 and 029 as 2.47 mg/kg, 1.11 mg/kg and 1.16 mg/kg. Non-cancer risk ranged as As (Nd -1.60 × 10^−3^), Cd (Nd–1.97 × 10^−4^), Cr (Nd-6.06 × 10^−6^), Hg (Nd–2.15 × 10^−4^), Ni (9.93 × 10^-6^–3.34 × 10^−5^) and Pb (Nd-4.36 × 10^−4^) while the cancer risk were As (Nd-1.63 × 10^−7^), Cd (Nd–4.45 × 10^−9^), Cr (Nd–1.56 × 10^−7^), Hg (Nd -1.53 × 10^−9^), Ni (1.50 × 10^−10^ –1.46 × 10^−9^) and Pb (Nd-8.82 × 10^−9^). The total cancer risk (TCR) and total non-cancer risk (TNCR) for all the heavy metals were in the range of 7.21 × 10^−13^–1.25 × 10^−10^ and 1.51 × 10^−7^–5.56 × 10^−5^ respectively. The TCR was below 1 × 10^−6^ -1 × 10^−4^ range while TNCR for heavy metals was below 1; the values established by US EPA. In conclusion, continuous consumption of locally Nigerian made painkiller drugs may expose the subjects to heavy metal toxicity.

## Highlights

• Nigeria locally made painkiller drugs contained As, Cd, Cr, Pb, Ni and Hg.
• Cancer and non-cancer risk values were below1 × 10^−6^ – 1 × 10^−4^ and one (1) US EPA standard value.
• True metal intake resulting from consumption of painkiller drugs were calculated.
• There exist a toxicological relationship between painkiller drugs and its heavy metal content.
• Painkiller drugs and its heavy metal content can act synergistically.

## Graphical abstract

**Figure:**
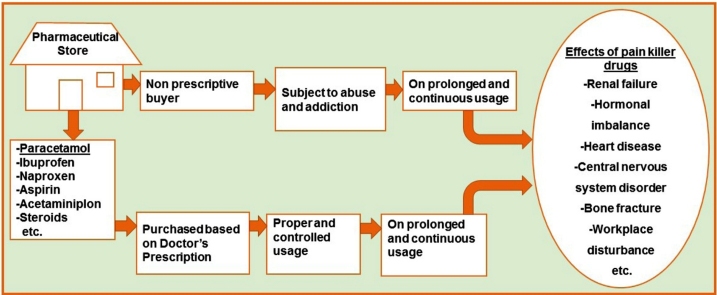

## 1 Introduction

Literacy statistics of 59.6 % cannot be regarded as a literate society in today’s modern world. Hence, it implies that higher population of Nigerians may not be aware of twin health hazards that may be caused by consumption of local analgesic drugs and the constituent heavy metal impurity. This is more worrisome by the non-existence of functional data on possible medical diagnostics result of ailments from painkiller drugs, heavy metal and other chemical constituents [1]. Rapidly growing and unregulated informal sector of the economy consisting of peasants, micro scale and several uncategorized occupation [2] is a sector of unspecialized skills where most jobs are at crude level and labour intensive, its intensity may result to body stress as most work place activities are still done manually against automation. The end result is somatic, neuropathic and dysfunctional pain, commonest amongst them are muscular pain and headache [3]. Treatment of pain is both physically and emotionally based but medications, have an overriding influence. The International Association for the Study of Pain defines it as encompassing damage to tissue and emotional fit associated with such damage [4]. A good number of pain tend to diminish on elimination of its cause while in some, it may persist despite removal of the inducer or more so when the cause is unknown [5]. Besides daily labourers on the lower rung of the economic indices, who are more prone to pain due to occupational and environmental hazard, most other citizens of different economic strata (professionals or semi-professionals) also experience pain. Pain is a form of medical condition with classification according to (a) “region of body involved- abdomen, lower limbs’’ (b) “system whose dysfunction may be causing the pain- nervous and gastrointestinal systems’’ (c) “duration and pattern of occurrence’’ (d) “intensity and time since onset and etiology” [6], though acceptance of this classification by medical professionals varies and were highly criticized [7]. Analgesics or painkillers are types of drugs used to sooth or eliminate pain [8]. They can in some conditions, through prolonged use or on addiction be a cause of attention or medical concern such as constipation [9], nausea, vomiting, drowsiness, itching, opioid-induced hyperalgesia [10], hormonal imbalance [11], work place disruption [12], erectile dysfunction, gastrointestinal effect, renal failure [13], increased proneness to accident [14], some of which has been ascribed to heavy metals. The six heavy metals - Arsenic (As), Cadmium (Cd), chromium (Cr), lead (Pb), mercury (Hg) and nickel (Ni) evaluated and found to be present, mimic and distort hormones and act as endocrine disruptors [15], thereby causing negative health outcome such as testicular injury [16], renal diseases, liver failure, diabetes mellitus, cancer [17], cardiovascular disease (CVD), blood pressure (BP) and atherosclerosis [18], bronchiole and alveoli inflammation [19], also implicated in skeletal and neurotoxic effect [20], these non-communicable diseases (NCDs) were reported to be in high occurrence than infectious diseases in Nigeria [21]. Overdose or frequent ingestion of painkiller drugs with the heavy metal contents may pose related risks as enumerated above. An often over looked health hazard is a possibility of association of pain drugs with heavy metals and even more due to synergistic effect and yet may not have been reported in Nigeria except for paediatric syrups [22,23]. Increased concentration of heavy metal in the environment aligns with the specific environmental media (soil, dust, water, and ambient air), different land use, and varied cultural usage of land resources, the metals are redistributed into human body via established pathways (skin, respiratory organ, and feeding and cultural habit such as geophagea). The human body may also through trophic level and industrialization be increasingly subjected to heavy metal overload. Locally manufactured painkiller drugs possibly laden with heavy metals are veraciously consumed in Nigeria. Non adherence to good manufacturing practices and inadequate raw materials processing may be possible sources of heavy metals in drugs. The main objectives of the current study is (a) determination of carcinogenic heavy metals (Hg, Cd, Ni, Cr, Pb and As) concentration in Nigerian locally made painkiller drugs, (b) evaluation of carcinogenic and non-carcinogenic health risk associated with ingestion of the locally made painkiller drugs and (c) establishment or evaluation of true metal intake.

## 2 Materials and method

### 2.1 Sampling and sample preparation

Thirty analgesic drugs, one packet for each drug type, were purchased from retail medicine outlets in Awka, Anambra State, Nigeria in September 2016 and were utilized in the analysis. Information on product labels and packets were noted. Aliquot samples of painkiller drugs were ashed and digested using Teflon lab wares previously cleaned in a high-efficiency particulate air (HEPA) filtered (class 100) in a clean laboratory to ensure non- metal impurities contamination. Cleaning of experimental wares was carried out in solutions using baths and in sequence (1 week each) and rinses (five per solution) employing three-stage process of detergent solution, deionised water cleaning, followed by 6 NHCl (reagent grade) solution with distilled-deionized water rinses and finally 7.5 N HNO_3_ (trace element grade) solution and ultra-pure water rinses. The lab wares were then dried using air polypropylene laminar flow exhaust hood; all these processes ensured quality assurance [22,23]. The samples were pulverized, divided and sieved to get finest particles. They were dry ashed on adding 10 g of each into a petri dish subjected to heat using hot plate at 200 °C, for 45 min, heated to char in the furnace at 500 °C. 1.5 g of ashed samples each were digested by addition of 10 mL conc. aqua regia (3:1; HCl: HNO_3_), then heated to dryness. 20 mL de-ionised water was added, stirred and filtered [24].

### 2.2 Sample analysis

The filtrates were made up in standard volumetric flask. Cadmium, lead, mercury, arsenic, nickel and chromium were assayed using Varian AA240 atomic absorption spectrophotometer according to American Public Health Association [25] with a detection limit of 0.001. The background level of blank was 0.001 mg/L.

The true metal intake using simple linear arithmetic mean according to Parkhurst [26], was calculated by multiplying contaminant level i.e., heavy metal level in the painkiller drug by the amount of painkiller drug intake per day. In all, the estimated or calculated levels of cadmium, lead, mercury, arsenic, nickel and chromium in the drugs were determined in selected few. 3000 mg (3 g) was assumed to be the average intake amount per day for each of the painkiller drugs. 1.5 g sample gave value of metals as depicted in Table 1. A prescription of 2 tablets x 3 per day (2 tablets each in the morning, afternoon and night, a tablet is 500 mg 2 × 3 = 3000 mg/1000 = 3 g). Example of true metal intake was calculated using four painkiller drugs coded 001, 002, 003 and 005.

**Table 1:** Heavy metal levels and label information of local manufactured painkiller drugs in Nigeria.

S/n	Product Code	Pharmacological Effect	Country of Manufacture	Nafdac. Reg. No	Batch No	Man.Date	Exp. Date	Heavy Metals (Mg/kg)					
								As	Cd	Cr	Hg	Ni	Pb
1.	01	(I)Analgesic (ii)Antipyretic	NIGERIA	04-0411	55571	10/14	10/19	0.350	ND	ND	0.470	0.125	ND
2.	02	(i)Analgesic (ii)Ant Ipyretic	NIGERIA	04-0633	0336	7/14	6/19	0.150	0.013	ND	0.108	0.100	ND
3.	03	(i)Analgesic (ii)Antipyretic	NIGERIA	04-7366	T621112	11/12	10/17	0.350	0.020	6.637	ND	0.434	ND
4.	04	(i)Analgesic (ii)Antipyretic	NIGERIA	B4-1774	493	10/14	9/18	0.250	ND	2.318	0.100	0.200	ND
5.	05	(i)Analgesic (ii)Antipyretic	NIGERIA	B4-1593	T4048	8/14	7/17	0.050	ND	ND	0.151	0.173	2.47
6.	060	(i)Analgesic (ii) Antipyretic	NIGERIA	A4-5653	349	8/14	7/17	0.150	0.065	ND	0.069	0.223	ND
7.	070	(i)Analgesic (ii) Antipyretic	NIGERIA	A4-0579	373	11/14	10/18	0.050	ND	ND	ND	0.270	ND
8.	080	(i)Analgesic (ii)Antipyretic	NIGERIA	A4-0536	9	2/14	1/18	0.250	0.033	0.194	ND	0.448	0.90
9.	090	(i)Analgesic (ii)Antipyretic	NIGERIA	04-0261	3417T	7/14	7/19	0.050	ND	ND	0.066	0.198	0.52
10.	010	(i)Analgesic (ii)Antipyretic	NIGERIA	04-2307	G0972	3/14	2/17	0.150	0.003	ND	ND	0.181	0.30
11.	011	(i)Analgesic (ii)Antipyretic	NIGERIA	04-7108	314	08/12	07/16	0.250	0.003	5.298	ND	0.228	0.59
12.	012	(i)Analgesic (ii)Antipyretic	NIGERIA	04-7312	983	7/13	6/18	0.250	0.011	1.298	ND	0.258	0.45
13.	013	(i)Strong Analgesic (ii)Strong Antipyretic	NIGERIA	04-7711	1000125	10/14	10/17	0.150	0.107	ND	ND	0.262	0.19
14.	014	(i)Strong Analgesic (ii)Strong Antipyretic	NIGERIA	A4-7884	B3006	2/13	1/16	0.250	ND	ND	0.005	0.111	ND
15.	015	(i)Strong Analgesic (ii)Strong Antipyretic	NIGERIA	04-2146	T54814	8/14	7/19	0.150	0.084	2.056	ND	0.046	0.05
16.	016	(i)Strong Analgesic (ii)Strong Antipyretic	NIGERIA	04-1860	0300346	4/14	3/19	0.050	0.072	ND	ND	0.143	0.15
17.	017	(i)Strong Analgesic (ii)Strong Antipyretic	NIGERIA	04-1233	3222535	12/12	12/17	ND	0.020	ND	ND	0.131	0.81
18.	018	(i)Strong Analgesic (ii)Strong Antipyretic	NIGERIA	B4-1238	M301	12/13	11/16	0.050	0.024	ND	ND	0.181	0.48
19.	019	(i)Strong Analgesic (ii)Strong Antipyretic	NIGERIA	04-4386	212090	04/13	03/17	0.050	0.048	ND	ND	0.162	0.40
20.	020	(i)Strong Analgesic (ii)Strong Antipyretic	NIGERIA	04-0005	154V	8/14	8/17	0.350	0.037	ND	ND	0.199	1.09
21.	021	(i)Strong Analgesic (ii)Strong Antipyretic	NIGERIA	04-1234	224083	8/14	8/18	0.150	0.115	ND	ND	0.140	0.54
22.	022	(i)Strong Analgesic (ii)Strong Antipyretic	NIGERIA	A4-0940	05	01/14	01/18	ND	0.087	0.449	ND	0.098	0.52
23.	023	(i) Analgesic (ii) Antipyretic (iii) Anti Inflammatory	NIGERIA	A4-2896	60	5/14	4/18	ND	0.057	0.163	ND	0.191	0.30
24.	024	(i) Analgesic (ii) Antipyretic (iii) Anti -00Inflammatory	NIGERIA	A4-7034	UPF303	1/13	1/16	0.050	0.060	ND	ND	0.145	0.70
25.	025	(i) Analgesic (ii) Antipyretic (iii) Anti Inflammatory	NIGERIA	A4-0486	23	7/14	6/18	0.150	0.021	0.479	ND	0.339	1.11
26.	026	(i) Analgesic (ii) Antipyretic (iii) Anti Inflammatory	NIGERIA	A4-3769	39	5/13	4/16	ND	0.094	ND	ND	0.174	0.91
27.	027	(i)Strong Analgesic (ii)Strong Antipyretic	NIGERIA	A4-0507	PCT-120	6/12	5/16	0.050	0.079	ND	0.003	0.179	0.76
28.	028	(i)Strong Analgesic (ii)Strong Antipyretic	NIGERIA	A4-6443	JI29	9/11	8/15	0.050	0.144	0.809	ND	0.161	0.75
29.	029	(i)Strong Analgesic (ii)Strong Antipyretic	NIGERIA	A4-8114	J4079	3/14	2/17	ND	0.058	ND	0.008	0.337	1.16
30	030	(i)Strong Analgesic (ii)Strong Antipyretic	NIGERIA	04-5278	40303	3/14	2/18	0.150	0.042	0.648	0.053	0.201	1.00

NAFDAC-National agency for food, drug administration and control; Man date- Manufacturing date; Exp. Date- Expiry date.

### 2.3 Human health risk assessment

(1) $$\begin{array}{cc} \left(\text{a}\right)\text{ Chronic Daily Intake}\left(\text{Carcinogens}\right) & \text{CD}{\text{I}}_{\text{ca}}=\frac{\text{CS}\times \text{ IR}\times \text{ EF}\times \text{ ED}\;\;\times \text{ CF}}{\text{BW}\times \text{ AT}} \end{array}$$

Where: CS depict exposure point concentration: mg/kg, IR is ingestion rate: 100 mg/d^−1^, EF is exposure frequency: 350 d/a, ED is exposure duration: 30a [27,28], BW is Body Weight: 70 kg [29], AT is averaging time for carcinogens is 365 × 70d, CF is Units conversion factor (10^-6^ kg mg^−1^) [30]

(2) $$\begin{array}{cc} \;\left(\text{b}\right)\text{ Chronic Daily Intake}\left(\text{Non-carcinogens}\right) & \text{CD}{\text{I}}_{\text{nca}}=\frac{\text{CS}\times \text{ IR}\times \text{ EF}\times \text{ ED}\;\;\times \text{ CF}}{\text{BW}\times \text{ AT}} \end{array}$$

Where: CS is exposure point concentration: mg/kg, IR is ingestion rate: 100 mg.d^−1^, EF is exposure frequency: 350 d/a, ED is exposure duration: 30a [27,28], BW is Body Weight: 70 kg [29], ^b^AT is averaging time for non-carcinogens = 365 x EDd [27,28], CF is Units conversion factor: 10^-6^ kg mg^−1^ [30]

(3) (c) Non-cancer risk (hazard quotient) HQ = CDI/RFDo

CDI is chronic daily intake (non-carcinogens) (mg kg^−1^ d^−1^), RFDo is chronic reference dose of the toxicant (mg kg^−1^ d^−1^); [RfD_O_ = Cd (0.0005); Cr (0.005); Ni (0.02); As (0.0003); Hg (0.003); Pb (0.0035)] [31]

Non-cancer risk (HQ) is the ratio of exposure to hazardous substances and translates into the total non-cancer risk (chronic hazard index) [HI] [32,33].

(4) TTHQ = THQ_As_ + THQ_Cd_ + THQ_Cr_ + THQ_Hg_ + THQ_Ni_ + THQ_Pb_

Addition of hazard quotient of each of the metals in all the drugs

(5) Hazard index due to heavy metals = ∑ CDI_k_ / RFD_k_

HI is the sum of more than one HQ for multiple substances or addition of hazard quotient of all the heavy metals in each drug. The acceptable value for the HI set by US EPA is < 1 [31]

(6) (d) Cancer Risk = CDI × SF

CDI is chronic daily intake (carcinogens) (mg kg^−1^ d^−1^), SF is slope factor (mg kg^−1^ d^−1^), calculated using the equation [34]:

(7) Cumulative cancer risk (CCR) = CR_As_ + CR_Cd_ +CR_Cr_ + CR_Hg_ + CR_Ni_ + CR_Pb_

This is linear summation of cancer risk of each heavy metal present in the 30 drug sample (if present) [1,28].

(8) Slope factor (SF) = 1/6 (ED)

But the total cumulative cancer risk can be calculated from [31]:

(9) $$Total\;cancer\;risk\;due\;to\;heavy\;metal\;=\overset{n}{\underset{k=1}{\sum }}\text{C }DI_{k}\;SF_{k}$$

This is the totality of cancer risk of all the heavy metals present in each drug sample.

The acceptable standard cancer risk value set by the US EPA is 1 × 10^−6^ - 1 × 10^-4^ [31]

## 3 Results

Table 1 contains information on 30 Nigerian locally manufactured painkiller drugs. 83.33 % of the samples contain arsenic, 80 % contain cadmium, 36 .67 % contain chromium while 33.33 % contain mercury. 83.33 %, 80 %, 36.67 %, 33.33 %, 100 %, and 80 % of all the drug sample contain near toxic levels of As^+3^, Cd^+2^, Cr^+6^, Hg^+2^, Ni^+2^and Pb^+2^. 19 (63.33 %) of the painkiller drugs contain nickel, arsenic and cadmium, while 100 % of the drugs contained nickel. 76.67 % of the drug samples contain lead. One (3.33 %) of the samples (code 030) contains all the five carcinogenic (As, Cd, Cr, Ni, Pb and Hg).

Table 2 shows that chronic daily intake (CDI) of the metals when considering their carcinogenic health effect. For arsenic, the range is Nd-2.055E-7; Cd (Nd-8.454E-8; chromium (Nd-3.897E-6); mercury (Nd-2.759E-7) while nickel ranged from 9.511E-8 – 2.630E-7 but lead (Nd -1.448E-6)

**Table 2:** Chronic daily intake (CDI) of heavy metals for carcinogenic effect of painkiller drugs.

S/N	Sample code	As	Cd	Cr	Hg	Ni	Pb
1.	010	2.055E-7	N D	ND	2.759E-7	7.339E-8	ND
2.	020	8.806E-8	7.632E-9	ND	6.341E-8	5.871E-8	ND
3.	030	2.055E-7	1.174E-8	3.897E-6	ND	2.548E-7	ND
4.	040	1.468E-7	ND	1.361E-6	5.871E-8	1.174E-7	ND
5.	050	2.935E-8	ND	ND	8.856E-8	1.016E-7	1.448E-06
6.	060	8.806E-8	3.816E-8	ND	6.751E-8	1.309E-7	ND
7.	070	2.935E-8	ND	ND	ND	1.585E-7	ND
8.	080	1.468E-7	1.937E-8	ND	ND	2.630E-7	5.266E-07
9.	090	2.935E-8	ND	1.139E-7	3.875E-8	1.162E-7	3.047E-07
10.	010	8.806E-8	1.761E-9	ND	ND	1.063E-7	1.750E-07
11.	011	1.468E-7	1.761E-9	ND	ND	1.339E-7	3.434E-07
12.	012	1.468E-7	6.458E-9	3.110E-6	ND	1.515E-7	2.660E-07
13.	013	2.935E-8	6.282E-8	7.620E-7	ND	1.538E-7	1.121E-07
14.	014	1.468E-7	ND	ND	2.935E-9	6.517E-8	ND
15.	015	2.935E-8	4.932E-8	ND	ND	2.701E-8	2.642E-08
16.	016	2.935E-8	4.227E-8	1.207E-6	ND	8.395E-8	8.748E-08
17.	017	ND	1.174E-8	ND	ND	7.691E-8	4.738E-07
18.	018	2.935E-8	1.409E-8	ND	ND	1.063E-7	2.824E-07
19.	019	2.935E-8	2.818E-8	ND	ND	9.511E-8	2.372E-07
20.	020	2.055E-7	2.172E-8	ND	ND	1.168E-7	6.411E-07
21.	021	8.806E-8	6.750E-8	ND	ND	8.219E-8	3.159E-07
22.	022	ND	5.108E-8	2.636E-7	ND	5.735E-8	3.041E-08
23.	023	ND	3.346E-8	9.570E-8	ND	1.121E-7	1.585E-07
24.	024	2.935E-8	3.523E-8	ND	ND	8.513E-8	4.685E-07
25.	025	2.935E-8	1.233E-8	2.812E-7	ND	1.990E-7	6.540E-07
26.	026	ND	5.519E-8	ND	ND	1.022E-7	5.366E-07
27.	027	2.935E-8	4.638E-8	ND	1.761E-9	1.051E-7	4.462E-07
28.	028	2.935E-8	8.454E-8	4.750E-7	ND	9.452E-8	4.403E-07
29.	029	ND	3.405E-8	ND	4.697E-9	1.978E-7	6.804E-07
30	030	2.935E-8	2.466E-8	3.804E-7	3.112E-8	1.180E-7	5.853E-07

Table 3 shows the chronic daily intake of non-carcinogenic effect. Arsenic is in the range of Nd-4.795E-7, cadmium (Nd-1.973E-7), chromium (Nd-9.092E-6), mercury (Nd-6.438E-7), lead (Nd-.379 E-6) and nickel (6.301E-8 -2.753E-6).

**Table 3:** Chronic daily intake (CDI) of heavy metal for non-carcinogenic effect of painkiller drugs.

S/N	Sample code	As	Cd	Cr	Hg	Ni	Pb
1.	010	4.795E-7	ND	ND	6.438E-7	1.718E-7	ND
2.	020	2.055E-7	1.781E-8	ND	6.438E-7	1.370E-7	ND
3.	030	4.795E-7	2.740E-8	9.092E-6	ND	5.945E-7	ND
4.	040	3.425E-7	ND	3.175E-6	1.370E-7	2.740E-7	ND
5.	050	6.849E-8	ND	ND	2.069E-7	2.370E-7	3.379E-06
6.	060	2.055E-7	8.904E-8	ND	9.452E-8	3.055E-7	ND
7.	070	6.849E-8	ND	ND	ND	3.699E-7	ND
8.	080	3.425E-7	4.521E-8	ND	ND	6.685E-7	1.229E-06
9.	090	6.849E-8	ND	2.658E-7	9.041E-8	2.712E-7	7.110E-07
10.	010	2.055E-7	4.110E-9	ND	ND	2.480E-7	4.082E-07
11.	011	3.425E-7	4.110E-9	ND	ND	3.123E-7	8.014E-07
12.	012	3.425E-7	1.507E-8	7.258E-6	ND	3.534E-7	6.206E-07
13.	013	2.053E-7	1.466E-7	1.778E-6	ND	3.589E-7	2.616E-07
14.	014	3.425E-7	ND	ND	6.849E-9	1.521E-7	ND
15.	015	2.055E-7	1.151E-7	ND	ND	6.301E-8	6.164E-08
16.	016	6.849E-8	9.863E-8	2.816E-6	ND	1.959E-7	2.000E-07
17.	017	ND	2.740E-8	ND	ND	1.795E-7	1.106E-06
18.	018	6.849E-8	3.288E-8	ND	ND	2.480E-7	6.599E.07
19.	019	4.795E-7	6.575E-8	ND	ND	2.219E-7	5.534E-07
20.	020	2.055E-7	5.068E-8	ND	ND	2.726E-7	1.496E-06
21.	021	ND	1.575E-7	ND	ND	1.918E-7	7.370E-07
22.	022	ND	1.192E-7	6.151E-9	ND	1.343E-7	7.096E-07
23.	023	6.849E-8	7.808E-8	2.233E-7	ND	2.616E-7	4.069E-07
24.	024	2.055E-7	8.219E-8	ND	ND	1.986E-7	1.093E-07
25.	025	ND	2.877E-8	6.562E-7	ND	4.644E-7	1.526E-06
26.	026	6.849E-8	1.288E-7	ND	4.110E-9	2.384E-7	1.252E-06
27.	027	6.849E-8	1.032E-7	ND	4.110E-9	2.452E-7	1.041E-06
28.	028	6.849E-8	1.973E-7	1.108E-6	ND	2.206E-7	1.027E-06
29.	029	ND	7.945E-8	ND	1.096E-8	3.661E-7	1.588E-06
30	030	2.055E-7	5.753E-9	8.877E-6	7.260E-8	2.753E-6	1.366E-07

Table 4 shows the non-cancer health risk of the heavy metals. That of arsenic is in the range of Nd-1.600E-3, cadmium (Nd-1.973E-4), chromium (Nd-6.06E-6), and mercury (Nd-2.150E-4), lead (Nd-4.360E-4) while nickel ranged from 9.930E-6 – 3.340E-5.

**Table 4:** Non- cancer risk (HQ) effect of heavy metals through painkiller drugs.

S/N	Sample code	As	Cd	Cr	Hg	Ni	Pb
1.	010	1.600E-3	ND	ND	2.150E-4	8.56E-6	ND
2.	020	6.850E-4	1.718E-5	ND	2.150E-4	6.850E-6	ND
3.	030	1.598E-3	2.740E-5	6.06E-6	ND	2.980E-5	ND
4.	040	1.140E-3	ND	2.12E-6	4.570E-5	1.370E-5	ND
5.	050	2.280E-4	ND	ND	6.900E-5	1.190E-5	4.229E-04
6.	060	6.850E-4	8.904E-5	ND	3.150E-5	1.530E-5	ND
7.	070	2.280E-4	ND	ND	ND	1.850E-5	ND
8.	080	1.140E-3	4.521E-5	ND	ND	3.340E-5	3.511E-04
9.	090	2.280E-4	ND	1.770E-7	3.010E-5	1.360E-5	2.031E-04
10.	010	6.850E-4	4.110E-6	ND	ND	1.240E-5	1.166E-04
11.	011	1.142E-3	4.110E-6	ND	ND	1.560E-5	2.326E-04
12.	012	1.140E-3	1.507E-5	4.840E-6	ND	1.770E-5	1.773E-04
13.	013	6.850E-4	1.466E-4	1.190E-6	ND	1.800E-5	7.474E-05
14.	014	1.140E-3	ND	ND	2.280E-6	7.610E-6	ND
15.	015	6.850E-4	1.151E-4	ND	ND	3.150E-6	1.760E-06
16.	016	2.280E-4	9.863E-5	1.88E-6	ND	9.800E-6	5.714E-06
17.	017	ND	2.740E-5	ND	ND	8.980E-6	3.160E-04
18.	018	2.280E-4	3.288E-5	ND	ND	1.240E-5	1.885E-04
19.	019	2.280E-4	6.575E-5	ND	ND	1.110E-5	1.581E-05
20.	020	1.60E-3	5.068E-5	ND	ND	1.360E-5	4.274E-04
21.	021	6.850E-4	1.575E-4	ND	ND	9.590E-6	2.106E-06
22.	022	ND	1.192E-4	4.100E-7	ND	6.720E-6	2.022E-04
23.	023	ND	7.808E-5	1.490E-7	ND	1.310E-5	1.163E-04
24.	024	2.28E-4	8.219E-5	ND	ND	9.930E-6	3.123E-05
25.	025	6.850E-4	2.877E-5	4.38E-7	ND	2.320E-5	4.360E-04
26.	026	ND	1.288E-4	ND	ND	1.190E-5	3.577E-04
27.	027	2.280E-4	1.082E-4	ND	1.370E-6	1.230E-5	2.974E-04
28.	028	2.280E-4	1.973E-4	7.390E-7	ND	1.030E-6	2.934E-04
29.	029	ND	7.945E-5	ND	3.650E-6	1.830E-5	4.357E-04
30	030	6.850E-4	5.753E-6	5.918E-7	2.420E-5	1.380E-5	3.903E-05

RfD_O_ = Cd (0.0005); Cr (0.005); Ni (0.02); As (0.0003); Hg (0.003); Pb (0.0035).

Table 5 shows cancer health risk values of the metals. Arsenic ranges from Nd-1.631E^−7^, cadmium (Nd-4.453E-9), chromium (Nd-1.562E-7), lead (Nd-8.823E-9), mercury (Nd-1534E-9) while nickel is in the range of 8.806E-10 – 1.416E-9.

**Table 5:** Cancer risk effect of heavy metals through painkiller drugs.

S/N	Sample code	As	Cd	Cr	Hg	Ni	Pb
1.	010	1.142E-9	ND	ND	1.534E-9	4.080E-10	ND
2.	020	4.893E-10	9.902E-11	ND	3.535E-10	3.264E-10	ND
3.	030	1.142E-9	1.522E-10	2.162E-8	ND	1.416E-9	ND
4.	040	8.156E-10	ND	7.562E-9	3.264E-10	6.523E-10	ND
5.	050	1.631E-10	ND	ND	4.928E-10	5.645E-10	8.045E-09
6.	060	1.631E-8	4.947E-10	ND	3.753E-10	7.273E-10	ND
7.	070	1.631E-7	ND	ND	ND	8.806E-10	ND
8.	080	8.156E-10	2.512E-10	ND	ND	1.461E-9	6.828E-09
9.	090	1.631E-10	3.950E-9	6.328E-10	2.154E-10	6.456E-10	3.950E-09
10.	010	1.631E-10	ND	ND	ND	5.906E-10	2.268E-09
11.	011	8.156E-10	2.284E-11	ND	ND	7.440E-10	4.453E-09
12.	012	8.156E-10	4.453E-9	1.728E-8	ND	8.417E-10	3.448E-09
13.	013	1.631E-10	2.284E-11	4.234E-9	ND	8.545E-10	1.454E-09
14.	014	8.156E-10	3.448E-9	ND	1.632E-11	3.621E-10	ND
15.	015	1.631E-10	8.373E-11	ND	ND	1.501E-10	3.415E-10
16.	016	1.631E-10	8.145E-10	6.706E-9	ND	4.664E-10	1.111E-09
17.	017	ND	ND	ND	ND	4.273E-10	6.145E-09
18.	018	1.631E-10	6.395E-10	ND	ND	5.906E-10	3.661E-09
19.	019	1.631E-10	5.480E-10	ND	ND	5.284E-10	3.075E-09
20.	020	1.142E-10	1.522E-10	ND	ND	6.489E-10	8.312E-09
21.	021	4.893E-10	1.827E-10	ND	ND	4.567E-10	4.095E-09
22.	022	ND	3.653E-10	1.465E-9	ND	3.196E-10	3.943E-09
23.	023	ND	2.816E-10	5.317E-10	ND	6.228E-10	2.261E-09
24.	024	1.631E-10	8.751E-10	ND	ND	4.730E-10	6.073E-10
25.	025	2.935E-10	6.623E-10	1.562E-7	ND	1.106E-9	8.479E-09
26.	026	ND	4.338E-10	ND	ND	5.678E-10	6.956E-09
27.	027	1.631E-10	4.567E-10	ND	9.790E-12	5.840E-10	5.706E-09
28.	028	1.631E-10	1.599E-10	2.635E-9	ND	5.252E-10	8.823E-09
29.	029	ND	7.156E-10	ND	2.611E-11	1.099E-9	8.823E-09
30	030	1.631E-10	6.012E-10	2.114E-9	1.730E-10	6.556E-10	7.590E-10

Table 6 is a linear summation of each target quotient of each metal in all the drug and were in the range of 1.859 × 10^−5^ (Cr) – 1.803 × 10^−2^ (As) while the cumulative summation of cancer risk of each heavy metal in all the drugs ranged from 3.523 × 10^−9^ (Hg) – 2.21 × 10^−7^ (Cr)

**Table 6:** Linear summation of target hazard quotient and cancer risk of all heavy in all drug sample.

Heavy Metals	As	Cd	Cr	Hg	Ni	Pb
TTHQ	1.803 × 10^−3^	1.724 × 10^−5^	1.859 × 10^−4^	6.378 × 10^−4^	4.017 × 10^−4^	4.745 × 10^−3^
CCR	1.881 × 10^−7^	1.987 × 10^−8^	2.210 × 10^−7^	3.523 × 10^−9^	1.970 × 10^−8^	1.001 × 10^−7^

Table 7 shows the total cancer and non-cancer health risk of five heavy metals in each drug. Total cancer risk ranged from 9.849E-13 – 1.251E-10 while that of total non-cancer risk is in the range of 6.757E-7 – 3.602E-5.

**Table 7:** Total cancer risk and total non-cancer risk effect of heavy metals through painkiller drugs.

S/N	Sample code	Total cancer risk $\sum_{k=1}^{n}\text{C}$DI_k_SF_k_	Total non-cancer (HI) risk $\sum_{k=1}^{n}\text{C}$DI_k_/RFD_k_
1.	001	9.247E -12	5.560E-5
2.	002	4.840E-12	1.489E-5
3.	003	9.708E-11	6.700E-6
4.	004	3.742E-11	2.307E-6
5.	005	3.661E-12	2.520E-6
6.	006	7.213E-13	2.858E-5
7.	007	2.088E-12	2.160E-5
8.	008	7.159E-12	4.959E-5
9.	009	3.036E-12	4.568E-7
10.	010	9.981E-12	2.234E-5
11.	011	4.711E-12	3.094E-5
12.	012	7.588E-11	5.238E-6
13.	013	2.240E-11	1.636E-6
14.	014	1.664E-11	2.152E-5
15.	015	1.763E-12	1.801E-5
16.	016	3.735E-12	1.513E-7
17.	017	9.849E-13	9.852E-6
18.	018	2.498E-12	1.661E-6
19.	019	2.546E-12	3.602E-5
20.	020	5.738E-12	2.483E-5
21.	021	3.966E-12	1.633E-5
22.	022	1.251E-10	5.711E-7
23.	023	2.680E-12	4.151E-7
24.	024	2.497E-12	2.416E-6
25.	025	1.160E-11	6.757E-7
26.	026	1.749E-12	1.810E-5
27.	027	3.027E-12	1.753E-5
28.	028	1.519E-11	1.048E-6
29.	029	3.950E-12	1.902E-5
30	030	1.034E-11	7.816E-6

RfD_O_ = Cd (0.0005); Cr (0.005); Ni (0.02); As (0.0003); Hg (0.003); Pb (0.0035).

## 4 Discussion

Impurity drug profile lies between organic and inorganics. Heavy metals are the main inorganic drug impurity which en-routes the bulk drugs and its intermediates via a number of processes [35]. Since drugs were not envisaged to contain toxic substances, safe levels of toxic As^+3^, Cd^+2^, Cr^+6^, Hg^+2^, Pb^+2^ and Ni^+2^ may not have been established [36], but a lot of data on toxic metals in pharmaceuticals abound [22,23]. Painkiller drugs are over-the-counter (OTC) medicines, consumed as non- prescriptions drugs and may be prone to abuse. Toxic heavy metals in drugs may portend danger to public health but in conjunction with heavy metal content in herbal remedies present a worst case scenario [35,37], as both enjoy high patronage in Nigeria. The values of Cd^+2^, Cr^+6^, Pb^+2^ and Ni^+2^ in this study (Table 1) did not vary significantly with same metals in paediatric syrup [22,23]. In further comparison, the heavy metal (Cd^+2^, Cr^+6^, Pb^+2^) values in drugs sold in India [38] were less than that in our study but differ from our work as they also examined for Ti, Fe, Cu, Zn and Co but did not evaluate for As^+3^, Ni^+2^ and Hg^+2^ as in this manuscript. Pharmaceutical effluents in India contain a near equal value of Cr^+6^, Pb^+2^, Cd^+2^ and Ni^+2^ in comparison with our work, although other metals were present in the waste effluent [39]. Furthermore many daily consumed products in Nigeria are laden with heavy metals as the value range in mg/kg of As (Nd – 0.350), Cd (Nd – 0.144), Cr (Nd – 6.637), Hg (Nd – 0.470), Ni (0.046 – 0.448) and Pb (Nd – 2.47) contained in painkiller drugs (Table 1) compares well with Cd (Nd – 2.45), Cr (Nd – 0.58), Ni (Nd – 4.13) and Pb (Nd – 1.08) in mg/l of paediatric syrup [22,23] but higher than Cd (Nd – 0.036), Ni (Nd – 0.050) and Pb (Nd – 0.036) mg/l of sachet or packaged water [40] and Cd (Nd – 0.081), Pb (0.002 – 0.075); Cd (Nd – 0.071) and Pb (Nd – 0.092) mg/l of canned and non-canned beverages [41]. Prolonged intake of painkiller drugs can be implicated in body organelles metal build up as a single prescription or non-prescription intake can expose a consumer to a minimum of two to a maximum of six metals as shown in Table 1. Modern and personal lifestyle such as smoking [42], intake of functional beverages [43], Nigerian local spices [44], street vended foods [45], skin infiltration of heavy metal via surface pores by application of skin enhancers, pedicure and manicure products [1,46], auto-paint dust [47,48], in addition to environmental and occupational exposures through intake of organic and inorganic air-driven suspensions [[49], [50], [51]], can add to the public heavy metal exposures, hence the human internal organs and systems may increasingly accumulate heavy metals. Chronic daily intake (CDI) for carcinogenic risk (Table 2) and non-carcinogenic health effects (Table 3) show similarity of values but with minor variations. The metals CDI values were in exponential range of 10^−6^ – 10^−9^, maximum values were 1.448 × 10^−6^, 3.897 × 10^−6^, 2.055 × 10^−7^, 8.454 × 10^−8^, 2.759 × 10^−7^ and 2.630 × 10^−7^ for Pb, Cr, As, Cd, Hg and Ni (carcinogens) (Table 2) while non-carcinogenic effect were in exponential range of 10^−6^ – 10^−9^ with maximum of 3.379 × 10^−6^, 9.092 × 10^−6^, 4.795 × 10^−7^, 1.973 × 10^−7^, 6.438 × 10^−7^ and 6.685 × 10^−7^ values of Pb, Cr, As, Cd, Hg and Ni (Table 3). Total target hazard quotient (TTHQ) is a linear summation of target hazard quotient (Table 4) of each heavy metal in all the drug samples (Table 6), from Table 6, TTHQ _As_ > TTHQ _Pb_ > TTHQ _Cd_ > TTHQ _Hg_ > TTHQ _Ni_ > TTHQ _Cr_, while hazard indices (HI) is the summation of hazard quotient (HQ) of all the heavy metals (As, Cd, Cr, Hg, Ni, Pb) in each of the drug sample. THQ, TTHQ or HI values greater than 1 is a chance for non-carcinogenic effect - diabetes, blood disease (increased blood pressure (HBP), stroke, hypertension), skeletal defect, paralysis [17,18,20], but when HI < 1, shows non- likelihood of occurring or acceptable risk for chronic system effect [[31], [32], [33]]. The results of our study (Table 4, Table 6) are all below 1 (the tipping point value) established by the US EPA for non-cancer health issues, but the probability of other sources or additive effect of all the metals in this study may trigger public health crises [42,51]. The cancer risk as depicted in Table 5 were well below 1 × 10^−6^ minimum value in a range of 1 × 10^−6^ – 1 × 10^−4^ US EPA standard value for cancer related issues as against 1 × 10^−12^ – 1 × 10^−7^ obtained in our study (Table 5). US EPA document states that cancer risk can be non-existent or insignificant when incremental life cancer risk (ILCR) value is lower than 1 × 10^−6^ but when ILCR equates to or surpasses 1 × 10^−4^ are important in risk study [29]. An incremental life time cancer risk (ILCR) above one in 10,000, meaning ILCR>10^−4^ is a situation for enquiry but when it is one - thousandth (1/1000) or greater (ILCR > 10^−3^) is a risk for further study. Linear addition of cancer risk or cumulative cancer risk (CCR) is a summation of cancer risk of each heavy metal in all the drug sample and it depicts CCR_Cr_ (2.210 × 10^−7^) as highest risk while CCR_Hg_ (3.523 × 10^−9^) as the lowest risk (Table 6) [34], but the total cancer risk is the addition of cancer risk of all metal per drug sample (Table7). The total cancer and non-cancer risks depicted in Table 7 shows exponential range of 9.849 × 10^−13^–1.251 × 10^−10^ for total cancer risk (TCR) and 6.757 × 10^−7^–5.560 × 10^−5^ for total non-cancer risk (TNCR). All the heavy metals per drug sample (As, Cd, Cr, Hg, Ni, Pb) present in this study are established to exhibit carcinogenic properties. Meanwhile, the values from Table 2, Table 3, Table 4, Table 5, Table 6, Table 7 were below comparative standards for carcinogenic and non-carcinogenic health effect of As^+3^, Cd^+2^, Cr^+6^, Hg^+2^, Ni^+2^, Pb^+2^. The values above may double or triple to a health crises emergency situation if the values of true or calculated metal intake were adopted in calculation of cancer and non-cancer risks (Table 8). The health hazards assumption from this work can be supported with the fact that tissues specimen of cancer patient showed As^+3^, Cd^+2^, Cr^+6^, Pb^+2^ and Hg^+2^ [52] with evidence of consumption of grains cultivated on heavy metal contaminated soil, it can therefore be correlated that the same metals found in painkiller drugs may likely have the same effect, placing this side by side with the fact that exposing human tissues to analgesics hinders sperm and egg maturity [53]. Ovaries exposed to Ibuprofen experience two- fifth drastic reduction in egg production and hastened menopause in female; a quartet reduction in sperm producing cells is observed when testicular tissue is subjected to either paracetamol or Ibuprofen [53,54]. Looking at the values of the true metal intake (Table 8), higher concentration or more of these metals can be ingested, most likely when they are abused, on prolonged usage or addiction. Estimated or the calculated true intake of As, Cd, Cr, Hg, Ni, and Pb shown in Table 8 was done by selecting the most frequently consumed painkiller drugs coded 001, 002, 003 and 005. The four most patronized painkiller drugs (coded 010, 02, 03, and 05) on exposure per day totalled -As (1.80 mg/kg), Cd (0.066 mg/kg), Cr (13.274 mg/kg), Hg (1.458 mg/kg), Ni (1.66 mg/kg), and Pb (4.940 mg/kg). These values raise serious concern. Summation of these findings with the fact that heavy metals, phthalates, parabens, phenols etc. and their precursors contained in some consumables-creams, lotions, toothpaste, fashion enhancers which are in high demand in Nigeria [1,46,54] cause early puberty in adolescents [55,56], assume hormonal action by manipulating activities of enzyme, inhibit fetal growth and may distort DNA causing permanent negative health issues [52,53,56]. Combined health hazards of painkiller drugs and heavy metals will definitely inhibit the health status of exposed victims. It therefore means that a toxicological relationship between the heavy metals and painkiller drugs exist (Table 9) and can exhibit twin negative health effect, as both can act synergistically (Table 9). Drugs or excipients are, on the whole, safe when there is compliance with prescription but adverse effects have been attributed to abuse or prolonged use. The necessary and accurate data on excipients in various preparations in Nigeria may not be readily available. Therefore, compulsory documentation of all excipients will help the consumers, physicians and health care givers to know the hidden ingredients in painkiller drugs and will improve their diagnostic skills. The product’s labels clearly show that all the drugs were registered and properly documented by the food and drug regulatory authority in Nigeria. It therefore, means that heavy metal monitoring may not be a priority for the regulatory authority. It is hoped that this work will raise the consciousness of food and drug regulatory authorities in Nigeria so as to include heavy metals in their standardization and routine monitoring.

**Table 8:** Calculation of true metal intake.

True metal intake	Calculation	Total intake of metal
As	2 mg x 0.350 + 2 mg x 0.150 + 2 mg x 0.350 + 2 mg x 0.050	1.80 mg/kg, As
Cd	2 mg x nd + 2 mg x 0.013 + 2 mg x 0.020 + 2 mg x nd	0.066 mg/kg, Cd
Cr	2 mg x nd + 2 mg x nd + 2 mg x 6.637 + 2 mg x nd	13.274 mg/kg, Cr
Hg	2 mg x 0.470 + 2 mg x 0.108 + 2 mg x nd + 2 mg x 0.151	1.458 mg/kg, Hg
Ni	2 mg x 0.125 + 2 mg x 0.100 + 2 mg x 0.434 + 2 mg x 0.173	1.664 mg/kg, Ni
Pb	2 mg x nd + 2 mg x nd + 2 mg x nd + 2 mg x 2.470	4.940 mg/kg, Pb

(Assumed painkiller drugs consumed per day was equivalent to two multiply by the amount used for the analysis, we randomly used drugs samples coded 001, 002, 003 and 005 for true intake calculation).

**Table 9:** Comparison of some hazardous effect of painkiller drugs with that of heavy metals.

S/No	Painkiller drug effect	References	Heavy metal effect	References
1	Constipation	9	Cd (abdominal cramps and vomiting)	57
2	Respiratory Depression	11	As (Respiratory effect-laryngitis, bronchitis rhinitis and Bracheobronchitis),	58
3	Hyperalgesia	10	Ni (Alveolar congestion and alveolar cell hyperplasia	59
4	Hormone imbalance	11	Cd, Cr^6+^, Ni, Hg (Endocrine disruptors and Metallohormones), As (chromosomal aberrations in peripheral lymphocytes	17,18
5	Gastrointestinal effect	13	As (Gastrointestinal effect	60
6	Renal problems	6	Cd (Renal effect) As (Renal effect)	17,19
7	Cardiovascular and congestive heart failure	10	As, Pb (Cardiovascular disease, myocardial infarction and artherial thickening, heart failure)	18,61
8	Fractures and Arthrities	6	Cd (Osteomalacia, osteoporosis and spontaneous fractures)	57
9	Central nervous system disorder	10	Hg (Neurological disorders, total damage to the brain and central nervous system (CNS)	62

(Some health effects caused by painkiller drugs compared well with those of heavy metals and can act synergistically).

## 5 Conclusion

The results have shown that 26 (86.67 %) of the drug samples contain at least four of the six carcinogenic metals (As, Cd, Cr, Ni, Pb and Hg) evaluated. All the drug sample (100 %) contain nickel while sample 030 contain all (100 %) heavy metals. Cadmium was contained in 80 % of the drug sample while Ni were contained in all of them. However, all the product’s labels contain regulatory agent’s numbers. This implies that ingestion of these painkiller drugs will expose an individual to the twin health effect of painkiller drugs and heavy metals, especially with cases of over dose, prolonged usage or on abuse. The chronic daily intake (CDI) values for metals ranged from 1 × 10^−6^ – 1 × 10^−9^ while carcinogenic and non-carcinogenic effect of the metals were below 1 × 10^−6^ and one (1). Heavy metals and painkiller drugs can act singly or synergistically to mimic hormones and may impair human health by distorting DNA, thereby causing permanent negative health desirability. The regulatory body in Nigeria should be encouraged to step up its monitoring activities to include heavy metals so as to reduce public health burden that may result when they are consumed.

## Author Statement

John Kanayocukwu Nduka, conceived, designed the work and carried out the laboratory work

Henrietta Ijeoma Kelle sourced the litratures

John Kanayocukwu Nduka and Henrietta Ijeoma Kelle did the calculations

Emeka Chima Ogoko reviewed the entire manuscript

All the author contributed financially for the work

## Research grant

We did not receive any research grant for this work

## Declaration of Competing Interest

The authors declare no conflict of interest.
